# The application of large language models in meteorology graduate research: current status, impact, and prospects

**DOI:** 10.1371/journal.pone.0347933

**Published:** 2026-04-24

**Authors:** Siguang Zhu, Honghui Li

**Affiliations:** 1 State Key Laboratory of Climate System Prediction and Risk Management/Key Laboratory of Meteorological Disaster, Ministry of Education/Collaborative Innovation Center on Forecast and Evaluation of Meteorological Disasters, Nanjing University of Information Science and Technology, Nanjing, China; 2 School of Atmospheric Sciences, NUIST, Nanjing, China; Virginia Polytechnic Institute and State University, UNITED STATES OF AMERICA

## Abstract

With the rapid development of generative artificial intelligence, large language models (LLMs) have gradually integrated into various fields, demonstrating significant potential, particularly in meteorological research. This study explores the current application, advantages, challenges, and future development trends of LLMs in the scientific work of meteorology graduate students at NUIST. Through surveys and case analysis, the study finds that LLMs are primarily applied in literature review, data processing, code development, and academic writing in meteorological research. The results show that LLMs significantly enhance research efficiency, particularly in code development and literature translation, saving considerable time for graduate students. However, challenges remain in areas such as the accuracy of professional knowledge, creative inspiration, and interdisciplinary integration. The study also reveals concerns over data security, academic integrity, and model limitations when using LLMs. Future applications of LLMs in meteorology need further optimization in terms of professional knowledge accuracy and data processing capabilities. This paper provides both theoretical support and practical guidance for the responsible integration of LLMs into meteorological research and education.

## Introduction

The integration of large language models (LLMs) into scientific research has revolutionized various fields, including meteorology, by offering new tools for data analysis, prediction, and decision-making. LLMs, such as OpenAI#39;s ChatGPT, have demonstrated remarkable capabilities in generating coherent and contextually relevant text, which has sparked significant interest in their application across diverse domains [1,2]. In the context of meteorological research, LLMs have shown potential in enhancing weather forecasting, climate modeling, and disaster prediction, particularly in regions prone to extreme weather events [3,4].

Meteorology, as a data-intensive and interdisciplinary science, presents unique challenges and opportunities for the integration of LLMs. Graduate students in meteorology often engage in complex tasks such as data interpretation, model simulation, and scientific writing, which could potentially benefit from the assistance of LLMs [5]. For instance, LLMs could aid in the summarization of vast amounts of meteorological literature, the generation of hypotheses based on existing data, and the drafting of research papers [6]. Moreover, the ability of LLMs to process and generate text in multiple languages could facilitate international collaboration and knowledge dissemination in meteorology [7].

Despite these potential benefits, the integration of LLMs into meteorological research is not without challenges. Concerns regarding the accuracy of generated content, the potential for bias in model outputs, and the ethical implications of using AI in scientific research must be carefully considered [8–10]. Additionally, the specialized nature of meteorological data and terminology may require tailored approaches to ensure that LLMs provide relevant and accurate assistance [11,12].

Recent advancements in AI-driven weather models, such as the FuXi and Pangu models, have demonstrated superior forecasting skills in predicting key meteorological variables, including 500 hPa geopotential height, 2 m air temperature, and precipitation [13,14,15]. These models have shown reliability in forecasting extreme weather events, such as tropical cyclones and heavy rainfall, with significant improvements in prediction accuracy and lead time [16]. Furthermore, the integration of AI models with traditional numerical weather prediction systems, such as WRF-ARW, has enhanced the ability to capture complex weather systems and improve forecast precision [17,18,19,20].

The application of LLMs in meteorology extends beyond numerical forecasting. Multimodal LLMs, such as CLLMate, have been developed to forecast weather and climate events by aligning meteorological raster data with textual event data, offering a comprehensive approach to event prediction [11,21]. This innovative framework leverages historical meteorological data and environmental news articles to construct a knowledge graph, enabling the prediction of open-set weather and climate events [22]. Such advancements highlight the potential of LLMs to transform meteorological research by providing intuitive and accurate insights into weather and climate phenomena [23].

In addition to their applications in meteorological research, LLMs have shown significant promise in academic research and writing [24,25,26]. The STORM system, for instance, has been developed to assist in the pre-writing phase of creating long-form articles, such as Wikipedia entries, by synthesizing topic outlines through retrieval and multi-perspective questioning [27]. This system simulates the pre-writing stage by discovering different perspectives on a given topic, simulating conversations with subject matter experts, and curating collected information to create a structured outline. The resulting articles are more organized and comprehensive, demonstrating the potential of LLMs to enhance the quality of academic writing [28].

LLMs can also be utilized in the context of academic paper reading and organization. Tools like LLM Research provide systematic literature reviews and detailed notes on key papers in the field of LLMs, helping researchers quickly grasp the core ideas and methodologies of important studies [29,30]. These resources are particularly valuable for graduate students and researchers who need to stay updated on the latest advancements in their field without spending excessive time on literature review [31].

In the realm of code writing for meteorological research, LLMs can assist in generating and optimizing code for data analysis and model simulation. For example, the MathCoder model has been developed to enhance mathematical reasoning by integrating code generation into LLMs, allowing for seamless code execution and iterative improvement of mathematical solutions [32,33]. This approach can be adapted to meteorological research, where complex numerical models and data analysis tasks often require sophisticated coding skills [34,35].

The integration of LLMs into meteorological research and academic writing offers numerous opportunities to enhance the efficiency and quality of scientific work. From improving weather forecasting models to assisting in the creation of comprehensive research articles, LLMs have the potential to revolutionize the way we conduct and communicate scientific research. However, it is crucial to address the challenges associated with accuracy, bias, and ethical considerations to ensure the responsible use of these powerful tools. As LLM technology continues to evolve, ongoing research and collaboration will be essential to fully realize its potential in meteorology and beyond.

## Methods

To explore the potential applications of large language models (LLMs) in meteorological research and academic writing, we conducted a survey targeting graduate students in meteorology at Nanjing University of Information Science and Technology (NUIST). The survey aimed to gather insights into how LLMs are currently being used, their perceived benefits and challenges, and the potential for future integration into meteorological research workflows.

### Survey design and distribution

The survey was designed using Tencent Questionnaire, a widely used online survey platform in China, known for its user-friendly interface and robust data collection capabilities. The survey consisted of both closed-ended and open-ended questions, covering topics such as the frequency of LLM usage, specific applications in research and writing, perceived advantages and limitations, and ethical considerations. The survey was distributed to graduate students enrolled in the meteorology program at NUIST between 2022 and 2024. Recruitment took place from March 1 to March 28, 2024. Participants were invited via email and social media platforms, and the survey remained open for a period of four weeks to ensure adequate response rates.

### Participants

The target population for this study was graduate students in meteorology at NUIST, specifically those enrolled between 2022 and 2024. This cohort was chosen because they are actively engaged in advanced meteorological research and are likely to be early adopters of new technologies such as LLMs. The survey was designed to capture a diverse range of perspectives, including students with varying levels of experience in using LLMs for research and academic writing.

### Data collection and analysis

Responses were collected anonymously through the Tencent Questionnaire platform, ensuring participant confidentiality. The data were exported in CSV format for further analysis. Quantitative data, such as frequency of LLM usage and perceived benefits, were analyzed using Microsoft Excel for descriptive statistics, including mean, median, and standard deviation. Python, along with libraries such as Pandas and Matplotlib, was used for more advanced statistical analysis and visualization, including the generation of bar charts, pie charts, and scatter plots to illustrate trends and correlations.

Qualitative data from open-ended questions were analyzed using thematic analysis. Responses were coded inductively to identify recurring themes and patterns. The coding process involved multiple iterations to ensure consistency and reliability. Themes were then categorized into broader categories, such as “Benefits of LLMs in Research,” “Challenges in LLM Integration,” and “Ethical Considerations.”

### Ethical considerations

The study was conducted in accordance with ethical guidelines for research involving human participants. Electronic informed consent was obtained from all participants prior to their participation in the survey. At the beginning of the online questionnaire, participants were presented with a consent form explaining the study#39;s purpose, the voluntary nature of their participation, and their right to withdraw at any time without penalty. Participants indicated their consent by checking an electronic consent box, and consent was automatically recorded and timestamped by the Tencent Questionnaire platform. All participants were graduate students aged 18 years or older, therefore parental or guardian consent was not required. Data were anonymized to protect participant identities, and all data were stored securely on password-protected servers.

### Limitations

While the survey provides valuable insights into the use of LLMs among meteorology graduate students at NUIST, there are some limitations to consider. The sample is limited to students at NUIST, which may not be representative of all meteorology graduate students globally. Additionally, the self-reported nature of the survey may introduce bias, as participants may overestimate or underestimate their use of LLMs. Future studies could expand the sample to include a broader range of institutions and incorporate observational data to complement self-reported measures.

### Conclusion

The methods outlined above provide a comprehensive approach to understanding the role of LLMs in meteorological research and academic writing among graduate students. By combining quantitative and qualitative data, this study aims to offer a nuanced perspective on the benefits, challenges, and ethical considerations associated with the use of LLMs in this specialized field. The findings will contribute to the growing body of literature on the integration of AI technologies in scientific research and education.

## Results

### Participant characteristics

**Fig 1:**
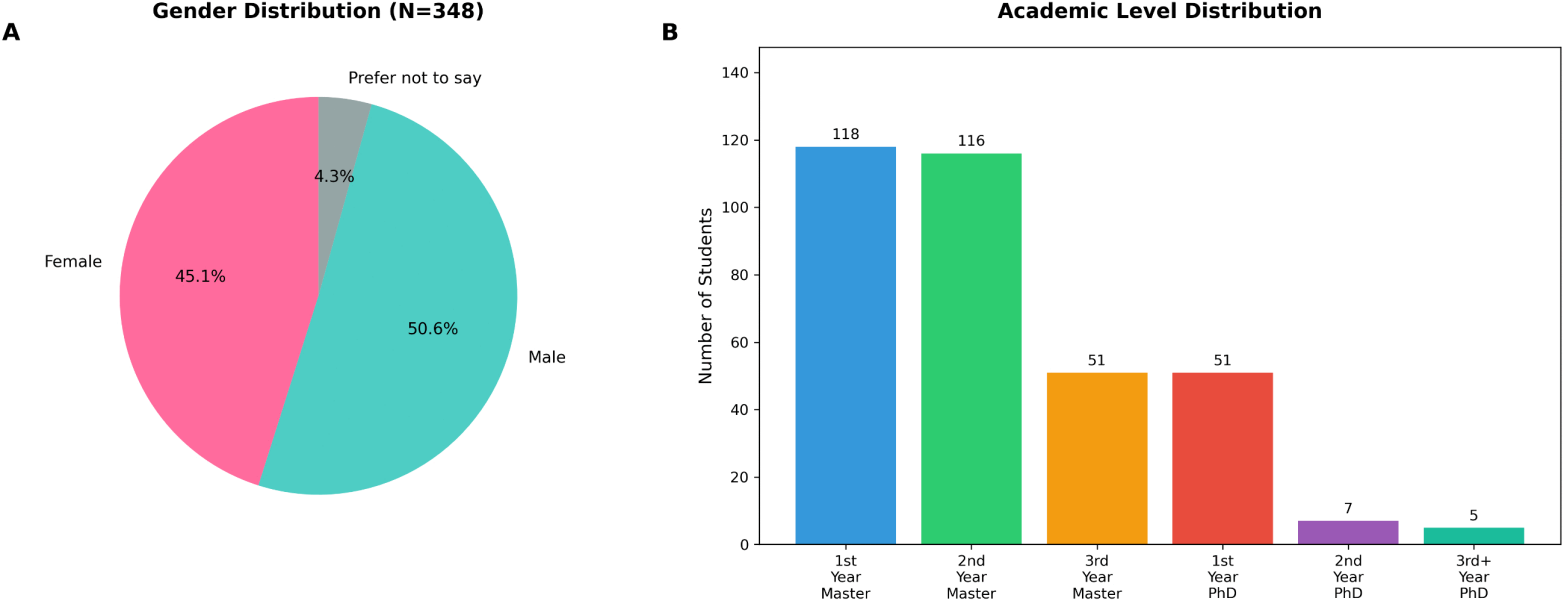
Gender and academic level distribution of survey participants (N = 348). **(A)** Pie chart showing gender composition with 45.1% female, 50.6% male, and 4.3% preferring not to disclose. **(B)** Distribution across academic levels, with master#39;s students comprising 81.9% of the sample.

A total of 348 graduate students completed the survey, representing a diverse cross-section of the meteorology graduate student population at NUIST. The sample included 157 female participants (45.1%), 176 male participants (50.6%), and 15 participants (4.3%) who preferred not to disclose their gender (Fig 1A).

The majority of respondents were master#39;s degree students (Fig 1B), with 118 first-year (33.9%), 116 second-year (33.3%), and 51 third-year students (11.7%). Doctoral students comprised 18.1% of the sample, including 51 first-year (11.7%), 7 second-year (2.0%), and 5 third-year or higher students (1.4%).Research areas represented in the sample included extreme weather prediction, tropical cyclone forecasting, climate change, mesoscale meteorology, ensemble forecasting, lightning prediction, land-atmosphere interactions, artificial intelligence applications, data assimilation, and paleoclimate modeling, reflecting the breadth of contemporary meteorological research.

### LLM awareness and usage patterns

**Fig 2:**
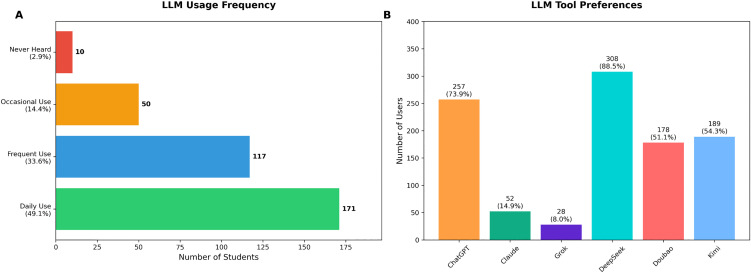
Large language model usage frequency and tool preferences. **(A)** Usage frequency categories showing 49.1% daily users and 33.6% frequent users. **(B)** Platform preferences with DeepSeek (88.5%) and ChatGPT (73.9%) as most popular tools.

The survey revealed high awareness and adoption of LLM tools among meteorology graduate students at NUIST. Only 10 participants (2.9%) reported never having heard of LLMs, while 97.1% had some level of familiarity and usage experience. Usage frequency was notably high, with 171 participants (49.1%) reporting daily use, 117 (33.6%) using LLMs frequently, and 50 (14.4%) using them occasionally (Fig 2A).

Regarding specific LLM platforms, DeepSeek was the most widely used tool, utilized by 308 participants (88.5%), followed by ChatGPT (73.9%), Kimi (54.3%), Doubao (51.1%), Claude (14.9%), and Grok (8.0%). Five participants (1.4%) reported using other LLM tools not listed in the survey options (Fig 2B).Weekly usage patterns showed intensive engagement, with 132 participants (37.9%) using LLMs more than 10 times per week, 84 participants (24.1%) using them 6–10 times per week, another 78 (22.4%) using them 3–5 times per week, 41 (11.8%) using them 1–2 times per week, and only 13 participant (3.7%) reporting no usage.

### Applications in meteorological research

**Fig 3:**
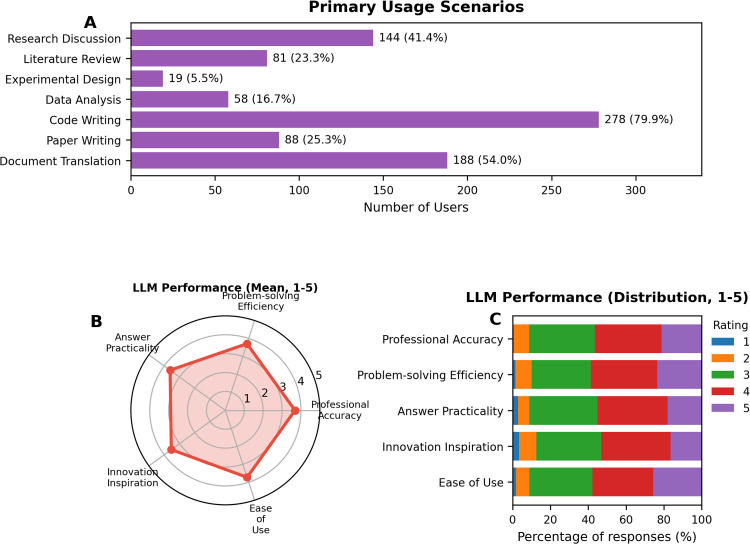
Application scenarios and performance evaluation of LLMs in meteorological research. **(A)** Horizontal bar chart of usage scenarios, with code writing as primary application (79.9%). **(B)** Radar chart of mean performance ratings (1-5 scale) across professional accuracy, efficiency, practicality, innovation, and ease of use. **(C)** Distribution of participant ratings across the five performance dimensions. Bars represent the percentage of responses on a 1–5 Likert scale.

The most common application of LLMs in meteorological research was code development and programming (Fig 3A), with 278 participants (79.9%) using these tools for coding purposes. This was followed by document translation (188 participants, 54.0%), research ideation and discussion (144 participants, 41.4%), paper writing (88 participants, 25.3%), literature review (81 participants, 23.3%), data analysis (58 participants, 16.7%), and experimental design (19 participants, 5.5%).

When asked about specific problems solved using LLMs in meteorological research, participants consistently mentioned code-related applications. Common responses included “code writing,” “code debugging,” “code modification,” “data processing,” and “program implementation.” Additional applications mentioned were literature translation, concept clarification, and research methodology discussion.

### Perceived benefits and performance evaluation

Participants identified three primary benefits of LLM usage in their research (limited to 3 selections): improving work efficiency (322 participants, 92.5%), optimizing code quality (250 participants, 71.8%), and getting new ideas (163 participants, 46.8%). Other benefits included solving technical problems (130 participants, 37.4%), improving English writing (123 participants, 35.3%), and deepening professional understanding (90 participants, 25.9%).

Performance evaluation across five dimensions revealed generally favorable ratings with moderate variability (Fig 3B–C):

- Ease of use received the most favorable ratings, with 89 participants (25.6%) giving a rating of 5 and 112 (32.2%) giving a rating of 4, representing the highest proportion of top-tier ratings among all dimensions.
- Problem-solving efficiency showed similarly strong performance, with 82 participants (23.6%) rating it 5 and 122 (35.1%) rating it 4, closely paralleling ease of use ratings.
- Practical utility of responses exhibited comparable ratings to problem-solving efficiency, with 129 participants (37.1%) rating it 4 and 125 (35.9%) rating it 3, though with a notably higher concentration in the middle range.
- Professional knowledge accuracy received more cautious evaluations, with ratings distributed across 123 participants (35.3%) at 4, 121 (34.8%) at 3, and 74 (21.3%) at 5, suggesting moderate confidence in domain-specific precision.
- Innovation inspiration showed the most distributed ratings, with 125 participants (35.9%) rating it 4, 103 (29.6%) rating it 3, and 74 (21.3%) rating it 5, indicating variable effectiveness in stimulating creative thinking.

To provide a clearer view of response variability across dimensions, Fig 3C presents the full distribution of ratings on the 1–5 Likert scale.

### Impact of academic stage on LLM usage

To investigate the potential influence of research experience on LLM adoption, we stratified the participants into four distinct academic stages: 1st-year Master’s (M1), 2nd-year Master’s (M2), 3rd-year Master’s (M3), and Doctoral students (PhD). The analysis revealed distinctive usage patterns associated with different phases of graduate training. First-year Master’s students reported the highest usage rate for code development (83.9%), followed closely by M2 (80.2%) and PhD students (79.4%), whereas M3 students reported the lowest usage (70.6%). In terms of literature processing, 3rd-year Master’s students demonstrated the highest reliance on document translation (66.7%), significantly surpassing M1 (50.0%) and PhD students (52.4%). Additionally, Doctoral students exhibited a high intensity of engagement, with 41.3% reporting heavy usage (more than 10 times per week), indicating that LLMs are deeply integrated into their daily research workflows.

### Challenges and limitations

**Fig 4:**
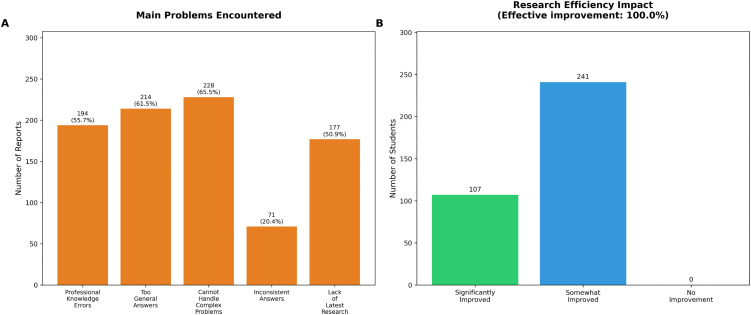
Challenges and research efficiency in LLMimplementation. **(A)** Frequency of reported problems, highlighting issues with complex problem handling and answer generality. **(B)** Research efficiency assessment, showing that 30.7% of participants reported significant improvement in productivity.

Participants identified several significant challenges when using LLMs for meteorological research (Fig 4A). The most frequently cited problems were inability to handle complex problems (228 participants, 65.5%), overly general answers (214 participants, 61.5%), and professional knowledge errors (194 participants, 55.7%). Other challenges included lack of access to latest research developments (177 participants, 50.9%) and inconsistent answers (71 participants, 20.4%).

Despite these challenges, all participants reported that LLMs had improved their research efficiency compared to traditional methods (Fig 4B), with 107 participants (30.7%) reporting significant improvement and 241 participants (69.3%) reporting some improvement.

### Improvement priorities and safety awareness

**Fig 5:**
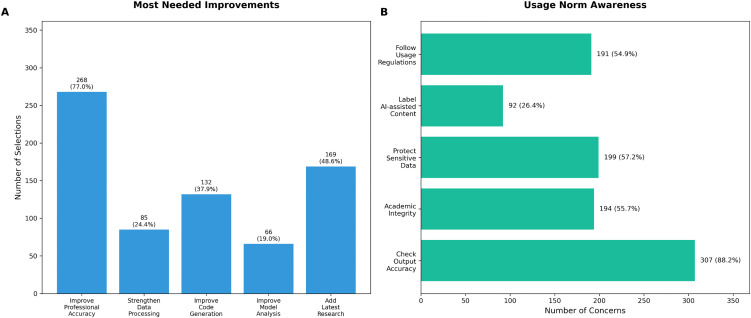
Improvement priorities and responsible usage practices of LLMsamong graduate students. **(A)** Priority ranking of improvements, with professional knowledge accuracy (77.0%), inclusion of latest research developments (48.6%), and code generation quality (37.9%) identified as top needs.**(B)** Responsible usage practices, showing that most participants (88.2%) reported verifying output accuracy.

When asked about areas most needing improvement (Fig 5A), participants prioritized enhancing professional knowledge accuracy (268 participants, 77.0%), followed by adding latest research developments (169 participants, 48.6%) and improving code generation quality (132 participants, 37.9%). Other priorities included strengthening data processing capabilities (85 participants, 24.4%) and improving model analysis capabilities (66 participants, 19.0%).

Regarding responsible usage practices (Fig 5B), most participants demonstrated awareness of important considerations: 307 participants (88.2%) reported checking output accuracy, 199 (57.2%) were concerned about protecting sensitive data, 194 (55.7%) paid attention to academic integrity, 191 (54.9%) followed usage guidelines, and 92 (26.4%) noted AI assistance in their work.

### Institutional attitudes and future perspectives

**Fig 6:**
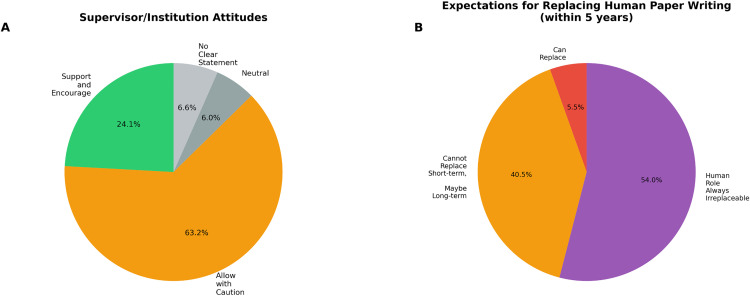
Institutional perspectives and future outlook on LLM integration in academic research. **(A)** Supervisor and institutional attitudes toward LLM usage, with the majority (63.2%) adopting a cautious but permissive stance. **(B)** Expert predictions on LLM's potential to replace human scientific writing within five years, with 40.5% believing replacement is unlikely in the short term but possible long-term.

Supervisors and research institutions generally showed supportive attitudes toward LLM usage (Fig 6A). 220 participants (63.2%) reported that their supervisors allowed but required cautious use, 84 (24.1%) reported encouragement and support, 23 (6.6%) indicated no clear institutional position, and 21 (6.0%) reporImprovement Priorities and Safety Awarenessted neutral attitudes. No participants reported institutional prohibition or discouragement of LLM use.

Regarding the potential for LLMs to replace human researchers in scientific writing within the next five years (Fig 6B), participants showed realistic expectations: 188 participants (54.0%) believed human roles would remain irreplaceable, 141 (40.5%) thought replacement was possible in the long term but not near term, and only 19 (5.5%) believed current replacement was possible.

## Discussion

Our findings reveal high adoption rates of LLM tools among meteorology graduate students at NUIST, with over 95% having experience with these technologies and nearly half using them daily. This rate is higher than the 40% usage reported in a study conducted at Northwestern University across medical, research, and educational contexts [1]. However, this difference should be interpreted with caution. Multiple factors may contribute to the observed variation, including disciplinary culture, institutional policies, temporal differences in data collection, and sample characteristics. Given the rapid evolution of LLM technologies between 2022 and 2024, broader societal acceptance may also partially explain the higher adoption observed in this study. Further research with controlled variables is needed to better understand the underlying drivers of these differences. The dominance of coding applications (79.9% of users) aligns with the computational nature of modern meteorological research, where students regularly work with numerical models, data analysis scripts, and visualization tools. This finding supports previous observations about LLMs’ particular utility in technical fields requiring programming skills [9,10,36].

The stratification analysis highlights how LLM utility evolves throughout the graduate training lifecycle. The high prevalence of coding assistance among early-stage students (M1) suggests that LLMs primarily serve as “scaffolding tools” for novices who are acquiring new programming skills (e.g., Python, NCL) and require support with syntax and debugging. As students progress to the final stage of their master#39;s degree (M3), the shift towards translation and literature review reflects the changing demands of their timeline—moving from skill acquisition to knowledge synthesis and thesis preparation. Doctoral students, maintaining high usage rates in both coding and high-frequency engagement, appear to leverage LLMs as versatile assistants for handling the complex, multifaceted tasks inherent in advanced research. Together, these findings suggest that LLM usage varies across stages of graduate training, ranging from technical scaffolding for early-stage students to knowledge synthesis support for more advanced researchers. Due to the relatively small number of PhD students in later stages, comparisons within doctoral subgroups should be interpreted cautiously. These findings should therefore be considered exploratory.

The universal report of efficiency improvements, with no participants reporting decreased productivity, suggests that LLMs have become valuable research tools despite their limitations. The primary benefits identified—improving work efficiency (92.5%) and optimizing code quality (71.8%)—indicate that students are successfully leveraging these tools for routine technical tasks, freeing time for higher-level analytical work.

However, the moderate ratings for professional knowledge accuracy (mean rating between 3 and 4) should not be interpreted as mere dissatisfaction. Instead, when viewed alongside our findings that 88.2% of participants actively verify output accuracy and 65.5% recognize the model#39;s inability to handle complex problems, these ratings indicate that students are actively calibrating their usage patterns. While 79.9% of students utilize LLMs for code development to enhance efficiency, the awareness of limitations, such as overly general answers (61.5%), ensures they maintain necessary skepticism. This ‘trust but verify’ approach demonstrates that meteorology graduate students are treating LLMs as auxiliary tools rather than authoritative sources.

The identification of significant challenges—particularly the inability to handle complex problems (65.5%) and overly general answers (61.5%)—demonstrates users’ awareness of current LLM limitations. These findings align with broader concerns about LLM capabilities in specialized scientific domains [2]. The high priority placed on improving professional knowledge accuracy (77.0%) suggests that students recognize the need for continued development in domain-specific applications.

The widespread practice of checking output accuracy (88.2%) indicates that students have developed appropriate verification strategies, which is crucial for maintaining research quality and academic integrity. This finding is encouraging given concerns about uncritical acceptance of AI-generated content in academic contexts.

The generally supportive institutional environment, with over 90% of participants reporting positive or neutral supervisory attitudes, suggests that the surveyed institution is adapting to technological change while maintaining appropriate oversight. The emphasis on cautious use rather than prohibition reflects a balanced approach to innovation and quality control. This supportive atmosphere contrasts with earlier findings from broader academic surveys, where institutional guidance was often reported as lacking or unclear [1,37]. The higher level of acceptance observed here may reflect the rapid evolution of AI policies over the past year, as well as the specific pragmatic needs of meteorology research where coding assistance is highly valued. However, as this study relied on data from a single program, it remains unclear whether this supportive atmosphere is specific to NUIST, characteristic of the meteorological discipline generally, or indicative of a broader trend across academia.

The realistic expectations about LLM capabilities in scientific writing—with 54.0% believing human roles remain irreplaceable—suggest that students maintain a healthy perspective on the complementary rather than replacement role of these technologies in research.

The findings suggest several implications for meteorology education and LLM integration, drawing on successful implementation strategies from other disciplines. First, formal training in effective LLM usage could help students maximize benefits while avoiding common pitfalls. Studies in educational technology indicate that structured AI literacy programs significantly improve student engagement and critical assessment capabilities. [8,38] Given the high usage rates and reported challenges, structured guidance on prompt engineering, output verification, and appropriate application domains could enhance educational outcomes. For instance, implementing ethical usage guidelines similar to those proposed for medical and general education can help mitigate risks associated with data privacy and academic integrity [10,39,40].

Second, the emphasis on coding applications suggests that LLM integration should be considered in computational meteorology curricula. This mirrors the successful integration of generative AI in engineering education, where it has been effectively utilized to enhance programming instruction and problem-solving skills. [9,41] As these tools become standard in research workflows, students will benefit from explicit instruction in their effective use.

Third, the development of meteorology-specific LLM tools or fine-tuned models could address the commonly cited issues of professional knowledge accuracy and domain complexity. Successful precedents in other specialized fields—such as the use of LLMs for medical licensing exams [42] and legal reasoning [43]—demonstrate the potential of domain-adapted models to support complex professional tasks. The suggestion by one participant to develop “AI specifically for earth sciences” reflects awareness of this need.

The survey results highlight a critical gap in current AI capabilities: over 77% of participants expressed a strong desire for LLMs to improve professional accuracy. This high demand indicates that generic LLMs, while useful for general tasks, are insufficient for the specialized needs of meteorological research. To address this, the development of meteorology-specific LLMs is essential. Such models should be trained on high-quality, domain-specific datasets—including historical climate data, real-time observational records, and peer-reviewed meteorological literature—to enhance terminology precision and reasoning reliability. Successful precedents in related fields demonstrate the potential of domain-knowledge-augmented LLMs to reduce hallucinations and inaccurate outputs. For example, chatClimate integrates external scientific knowledge to improve response accuracy [44]. However, the transition to domain-specific applications also presents challenges. Previous research has emphasized that ensuring the trustworthiness of these tools requires rigorous scrutiny of biases and inaccuracies, as well as robust evaluation protocols [45]. Therefore, future development must prioritize not only data integration but also the establishment of robust evaluation frameworks to transform LLMs from general assistants into reliable scientific instruments.

This study has several limitations that should be acknowledged. The sample is limited to a single institution and may not represent the broader population of meteorology graduate students globally. The self-reported nature of the data may introduce bias, and the cross-sectional design limits our understanding of usage pattern evolution over time.

Future research could expand to multiple institutions across different countries and regions, to better disentangle the specific influences of disciplinary culture versus institutional policies, and include faculty perspectives to provide a more comprehensive view of LLM integration in meteorological research and education. Longitudinal studies could track how usage patterns and attitudes evolve as these technologies develop and mature.

While this study focuses on graduate students, the implications for undergraduate education warrant specific attention. unlike graduate researchers who possess a foundational understanding of meteorological principles, undergraduate students may lack the necessary domain expertise to critically evaluate LLM outputs, making them more susceptible to “hallucinations” and conceptual errors. Therefore, university policies regarding AI usage for undergraduates likely require more structured guidance and strict verification protocols compared to those for graduate researchers. Future studies should extend this scope to the undergraduate level to compare usage patterns and educational impacts across different training stages.

## Conclusion

This study provides preliminary evidence regarding LLM usage among meteorology graduate students through a detailed case study at NUIST, revealing high adoption rates, substantial efficiency benefits, and sophisticated usage strategies. While students have successfully integrated these tools into their research workflows, they maintain realistic expectations about capabilities and limitations.

The findings suggest that LLMs have become valuable research tools in meteorology, particularly for coding and routine technical tasks. However, the persistence of challenges related to professional knowledge accuracy and complex problem-solving indicates continued need for human expertise and critical evaluation.

The generally supportive institutional environment and students’ awareness of quality control practices suggest that the studied cohort of meteorology researchers is successfully navigating the integration of these powerful new tools while maintaining research integrity. As LLM technology continues to advance, ongoing dialogue between students, faculty, and technology developers will be essential to maximize benefits while addressing persistent challenges. However, as this study is based on a single-institution case study, further multi-institutional research is required to determine whether these patterns can be generalized to other academic contexts.

The development of domain-specific tools and training programs could further enhance the value of LLMs in meteorological research and education. By building on the foundation of responsible usage practices already evident among current students, the meteorology community can continue to leverage these technologies effectively while preserving the critical thinking and specialized expertise that remain essential for advancing atmospheric science.

## Supporting information

S1 Data Raw questionnaire data. Original survey responses used in this study.
(CSV)
